# Evolutionary pressures on microbial metabolic strategies in the chemostat

**DOI:** 10.1038/srep29503

**Published:** 2016-07-06

**Authors:** Meike T. Wortel, Evert Bosdriesz, Bas Teusink, Frank J. Bruggeman

**Affiliations:** 1 Systems Bioinformatics, VU University, Amsterdam, De Boelelaan 1087, 1081 HV, The Netherlands

## Abstract

Protein expression is shaped by evolutionary processes that tune microbial fitness. The limited biosynthetic capacity of a cell constrains protein expression and forces the cell to carefully manage its protein economy. In a chemostat, the physiology of the cell feeds back on the growth conditions, hindering intuitive understanding of how changes in protein concentration affect fitness. Here, we aim to provide a theoretical framework that addresses the selective pressures and optimal evolutionary-strategies in the chemostat. We show that the optimal enzyme levels are the result of a trade-off between the cost of their production and the benefit of their catalytic function. We also show that deviations from optimal enzyme levels are directly related to selection coefficients. The maximal fitness strategy for an organism in the chemostat is to express a well-defined metabolic subsystem known as an elementary flux mode. Using a coarse-grained, kinetic model of *Saccharomyces cerevisiae*’s metabolism and growth, we illustrate that the dynamics and outcome of evolution in a chemostat can be very counter-intuitive: Strictly-respiring and strictly-fermenting strains can evolve from a common ancestor. This work provides a theoretical framework that relates a kinetic, mechanistic view on metabolism with cellular physiology and evolutionary dynamics in the chemostat.

How do selection and phenotypic adaptation shape cellular physiology in a particular environment? Long-term adaptation experiments with microorganisms are an interesting approach to study this question. Chemostats—continuous cultivation devices that feed nutrients at a constant rate in a fixed volume, thereby forcing the steady-state specific growth rate of the cells to match the medium dilution rate—are well suited for such experiments, especially after the introduction of small-scale chemostats that reduce the running costs^1^,^2^,^3^,^4^. In this paper we will focus on the metabolic adaptation of microorganisms that evolve in such a chemostat environment.

Dean, Dykhuizen and Hartl were among the first to study the relation between fitness, protein expression and metabolic fluxes in a chemostat setting^5^,^6^. Their work was guided by insights from metabolic control analysis (MCA)^7^. This theory predicts that the influence of an enzyme on a metabolic flux decreases with its expression level, and that the flux through a metabolic pathway is a concave, hyperbolic function of the enzyme activity. Given this, they reasoned that increasing enzyme activity is accompanied by diminishing returns, eventually leading to selective neutrality when an enzyme is highly expressed^5^. They confirmed these expectations experimentally for *β*-galactosidase and *β*-galactoside-permease activity^6^,^8^,^9^.

However, MCA does not take the fitness cost of enzyme expression into account, which increases with expression level. The limited biosynthetic capacity of a cell forces increased expression of a particular enzyme to go at the expense of other enzyme concentrations^10^, which will at some point cause a decrease in fitness with increasing expression. This is to be expected when the increase in costs of protein expression becomes larger than its increased benefit^11^. From a large number of studies it has since become evident that such costs plays an important role in determining fitness. For instance, expression of non-functional proteins reduces growth rate in batch cultures^10^,^12^,^13^,^14^ and fitness in chemostats^15^,^16^. Moreover, a number of studies, with different organisms and different metabolic systems, show that overexpression of enzymes reduces fitness^17^,^18^,^19^,^20^,^21^. Enzyme costs appear to arise from the process of making the enzyme^16^,^22^. Enzymes that are overexpressed, and therefore have some appreciable fraction that is unused, are expected to cause a fitness reduction, rather than being fitness-neutral.

The fitness benefit and cost of protein expression suggests the existence of an (environment-dependent) optimal expression level where the difference between the benefit – the biochemical activity – and cost – the resource consumption during expression – is maximized^11^. Indeed, in a laboratory-evolution experiment, *Escherichia coli* attained predicted, environment-dependent optimal expression levels within 500 generations^23^. The important role of protein costs on physiology is perhaps best exemplified by the covariation of the proteome of *E. coli* with growth conditions, where the expression levels of whole sectors of metabolism are tuned to the environmental requirements^24^. For instance, the ribosomal protein fraction increases linearly with growth rate in *E. coli*^10^,^25^; likely because the ribosome concentration is precisely tuned to the prevailing conditions to prevent overexpression^26^,^27^. Another example is the switch from respiration to fermentation with increasing substrate availability, which is observed in many micro-organisms. Molenaar *et al.* hypothesized that this was the result of the relatively high enzyme-investment required for respiration^28^. Recently, Basan *et al.* tested this hypothesis for the overflow metabolism in *E. coli*^22^. Their results were in agreement with this hypothesis and ruled out a number of other ones, such as limitations in respiratory capacity or cytoplasmic membrane area. These insights raise the question: which enzymes should be expressed, and to what concentration, to achieve fitness maximization?

We recently addressed these questions for cells growing in a constant environment^11^,^29^. We showed that an optimal metabolic strategy must be an elementary flux mode (EFM)^29^, which is a minimal, steady-state ‘route’ through a metabolic network. An EFM represents a ‘pure’ metabolic strategy, e.g. fermentation or respiration, but not respiro-fermentation. We also found that the optimal enzyme concentration is proportional to the influence it exerts on the flux, its flux control coefficient^11^ (Also see refs 7, 30 and 31). This flux control coefficient we could relate to the selection coefficient and the fitness costs and benefits of enzyme expression.

Because a feedback occurs from the physiology of the cell to the environmental conditions in a chemostat, the existing theoretical understanding of the evolution in a constant environment^11^,^29^ cannot immediately be extrapolated to the chemostat. Our aim is therefore to formulate a theory of optimal metabolic strategies in a chemostat, expressed in terms of kinetics and costs of metabolic enzymes. We will extend the theory from constant conditions to chemostat conditions, and show the theoretical possibilities for metabolic evolution in the chemostat. Furthermore, we develop a modeling strategy to perform evolutionary simulations of a ‘self-replicator model’^28^ of metabolism and growth of *Saccharomyces cerevisiae* in a chemostat (see ref. 32 for another approach to coarse grained models, incorporating details of translation and ribosome competition, more explicit trade-offs and less metabolic detail). We use this model to illustrate how selection shapes the decision between respiratory and fermentative metabolism. We find that negative frequency-dependent selection causes the evolutionary-stable coexistence of a purely fermenting and a purely respiring strain, and that these strategies can evolve from a common ancestor that does both.

## Methods

### Model description

In this section we will briefly discuss how we simulate metabolism and growth of a coarse-grained self replicator model of yeast in a chemostat environment. It is explicitly not our intention to provide an as-realistic-as-possible model of *S. cerevisiae*. Rather, we use a simplified model to focus on how evolutionary pressures relating to the protein economy affect the ‘choice’ between respiration and fermentation, specifically when taking the feedback of the cellular physiology on the conditions in the chemostat into account. For details and a mathematical description we refer to the Supplementary Information S2.

#### A self replicator model of yeast

The basic concept behind our model is that it is a self-contained representation of cellular growth. We model the cell as a self-replicator, with a focus on metabolism and protein synthesis. The ribosomes synthesize all proteins required for growth, including themselves. (DNA and RNA synthesis are not included in the model). The model is coarse grained, meaning that a large number of reactions are lumped into a single rate equation.

**Figure 1:**
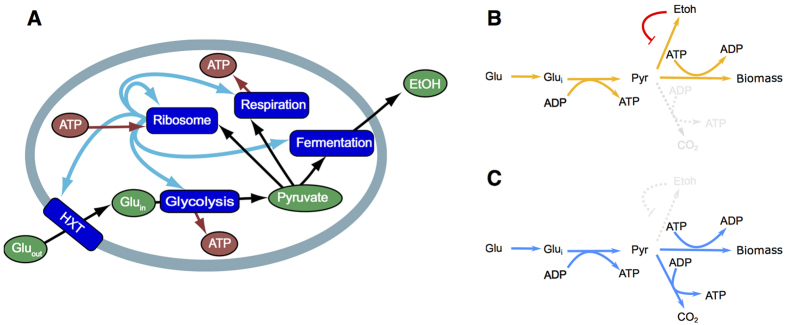
A self-replicator model of a *Saccharomyces cerevisiae* cell. This coarse-grained model captures the main metabolic pathways of a *S. cerevisiae* cell. Glucose is transported into the cell by the *HXT*-transporter, and is subsequently metabolized to pyruvate by glycolysis. Next, pyruvate can serve as a precursor for biomass formation by the ribosome, or it can be further metabolized through either fermentation or respiration, the latter yielding additional ATP. The model has two elementary flux modes, a fermentative mode (**B**) and a respiratory mode (**C**).

The model consists of a glucose transporter, glycolysis, a fermentation pathway, a respiration pathway, and ribosomes (Fig. 1A). The transporter transports glucose into the cell. Next, it is metabolized by glycolysis, yielding two pyruvate and two ATP. Pyruvate and ATP are both requires for protein synthesis by the ribosomes, but in a ratio of 2.4 ATP per pyruvate. As a consequence, more ATP needs to be generated to balance its production and consumption. This is done by the additional uptake of glucose that is metabolized in two different ways: fermentation or respiration. In fermentation, the additional glucose is metabolized to pyruvate after which it is discarded in the form of ethanol. Alternatively, in respiration, pyruvate is completely metabolized to CO_2_, which generates an additional 9 ATPs per pyruvate. This means that our model has two EFMs, which we refer to as the respiratory and the fermentative strategy (Fig. 1B,C). A mixed strategy uses a combination of these EFMs, and is characterized by the respiratory ratio, the flux towards respiration over the total flux towards respiration and fermentation. We refer to cells employing a pure respiratory or fermentative strategy as respirers and fermenters, respectively. Fermentation and respiration differ in their protein requirement. While respiration generates more ATP per glucose molecule, it is more costly than fermentation because it requires more proteins per unit flux.

Transport of glucose is modeled as facilitated diffusion, which ensures that product inhibition still affects the rate, even at high substrate concentrations. All other reactions are described by Michaelis Menten rate equations with product inhibition, meaning that they become saturated at high substrate concentrations. Importantly, in our model extracellular ethanol inhibits the fermentative flux, but not the respiratory flux. This is based on experimental results that suggest a stronger inhibition on (partially) fermenting strains^33^. Moreover, ethanol slows down the glycolytic flux in *S. cerevisiae* after a glucose pulse, a regime in which the yeast mainly ferments^34^.

The biosynthetic flux – the rate at which ribosomes synthesize the required proteins – sets the self-replication rate, which we interpret as the specific growth rate. The enzyme concentrations are set by the fraction of ribosome dedicated to the synthesis of that enzyme, and the dilution rate. The higher the concentration of an enzyme, the more cellular resources – ribosomes, precursors and ATP – are required to maintain that concentration. This makes overexpression costly. On the other hand, if the concentration of an enzyme is too low, it becomes a bottleneck and starts limiting the biosynthetic flux. In other words, maximizing the growth rate requires each enzyme to attain an optimal concentration. What these optimal concentrations are depends on the extracellular glucose and ethanol concentration.

The parameters of our model are as much as possible based on literature. However, the *k*_*cat*_-values of pathways, i.e. the maximal turnover of a pathway per unit enzyme per unit time, were not available. We therefore estimated their relative value based on the number and size of the different enzymes in the pathway; Pathways with many or large enzymes have a lower *k*_*cat*_. Subsequently, by multiplying all relative *k*_*cat*_ s with the same factor, we fitted their absolute value such that the model had a realistic maximal growth rate. While we could make a realistic estimate for the other modules, the costs for respiration, such as the maintenance of mitochondria and damage due to reactive oxygen species, was difficult to assess. We fitted the cost of the respiration module to ensure a switch between optimal strategies from respiration to fermentation at intermediate glucose concentrations of 0.265 *h*^−1^, which has been observed in experiments.

#### Chemostat model

In a chemostat, medium enters and leaves the vessel at a fixed flow rate (thereby keeping the volume constant), which determines the dilution rate *D*. The medium is composed such that one nutrient will become growth-rate limiting; in our model, this is glucose. The glucose concentration in the vessel reaches a concentration where the growth rate equals the dilution rate. An experimentalist can therefore set the growth rate by changing the dilution rate. We modeled the concentrations of biomass, glucose and ethanol in the chemostat with differential equations describing in- and outflow, growth, nutrient uptake and ethanol production. We used the self-replicator model discussed above to calculate the growth rate of the cells as a function of enzyme levels and of the glucose and ethanol concentration in the chemostat. In this way, we can relate the kinetics of metabolism and growth to the dynamics of biomass density in the chemostat.

Cells that ferment produce ethanol, which inhibits fermentation. Since more fermenting cells produce more ethanol, our model contains negative frequency-dependent selection. The steady-state biomass density and the glucose and ethanol concentration in the chemostat are therefore all interdependent. Ultimately, they are set by the dilution rate, the glucose concentration in the feed and the kinetic properties of the organisms in the chemostat.

## Results

### Evolutionarily stable strategies in a chemostat must be elementary flux modes

In a chemostat, the steady-state growth rate of cells equals the dilution rate that is set by the experimenter. The growth rate of a cell depends on the concentrations of the limiting nutrient and inhibiting compounds, which are all variable in a chemostat. Evolution in a chemostat does therefore not simply select for growth-rate maximization at fixed nutrient levels, and it is not straightforward to define what an optimal strategy entails. Moreover, the optimal phenotype, carrying out the optimal strategy, should be evolutionarily stable, such that a mutant with a different strategy (or with different enzyme concentrations) is not able to invade. Since a mutant can only invade the chemostat by growing faster than the dilution rate, selection is still ultimately mediated through differences in growth rate.

In a previous paper, we have presented a formal and extensive proof that, in a constant but otherwise arbitrary environment, the highest specific growth rate is always achieved by an EFM^29^. An EFM is a metabolic subnetwork that can attain a steady state, carries a flux in a thermodynamically-feasible direction, none of its enzymes can be removed without violating the steady-state requirement, and it has one independent flux (only one flux value needs to be known to determine all flux values at steady state). The intuition behind this proof is as follows: Suppose a cell has two parallel metabolic pathways (EFMs) to generate for instance ATP. The rate of ATP-synthesis per amount of protein invested in these pathways will not be the same. In other words, the ‘return on investment’ differs between these pathways. This implies that the total return on investment for the cell can be enhanced by taking resources away from the pathway with the lower return on investment, and instead invest them in the more productive pathway. A metabolic network typically has many EFMs, and which particular one is optimal depends on the environmental conditions and metabolic enzyme kinetics^29^.

Although a chemostat is designed to operate at steady state, it is actually not a constant environment in any evolutionary sense, as changes in microbial physiology affect, for instance, the concentration of substrates and inhibitors in the vessel. However, as selection in the chemostat is ultimately mediated through differences in growth rate, any optimal strategy in a chemostat must be an EFM. This can be shown by a ‘Gedankenexperiment’. Suppose that the optimal strategy is not an EFM. When the vessel is in steady state and hence the environment is constant, there must be an alternative strategy that can grow faster under the prevailing conditions, because under such constant conditions the maximal-growth-rate strategy is an EFM. A mutant employing this strategy will be able to invade and hence, a non-EFM strategy can be invaded and therefore cannot be an optimal strategy. We will illustrate some implications of this result with a coarse-grained whole cell model of yeast in a chemostat environment (Fig. 1). Our self-replicator model has two EFMs: A purely fermentative strategy and a purely respiratory strategy. The reasoning above implies that a respiro-fermentative strategy cannot be an optimal strategy, as this is not an EFM but a mixture of two. A respiro-fermentative strategy can always reach a higher growth rate by reallocating proteins from respiration to fermentation or visa versa, to the mode that gives the highest return on investments under the current conditions. This results simplifies finding the potential evolutionary steady states in a chemostat, because it greatly limits the set of possible solutions.

### The relation between enzyme concentrations and selection coefficients

In this section, we study what the optimal enzyme concentrations within an EFM are and we quantify the fitness effects of deviations from optimality. Selection between two species or mutants in a chemostat is typically quantified by the selection coefficient 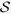, which is the time derivative of the ratio of biomass densities^35^. For convenience, it can be normalized by the dilution rate *D*. We are specifically interested in the effect of changes in enzyme levels. The selection coefficient 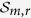 of a mutant *m*, with a (infinitesimal) perturbation in the concentration of enzyme *i* compared to a resident *r*, *e*_*i*_ → *e*_*i*_ + *δe*_*i*_, is given by (cf. Supplementary Information S1)


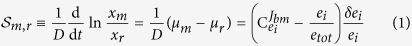


where *x* is the biomass concentration, *μ* the specific growth rate, *e*_*tot*_ is the total enzyme concentration in a cell, and 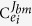 is the flux control coefficient of enzyme *i* on the rate of biomass formation *J*_*bm*_. The flux-control coefficient – a concept from Metabolic Control Analysis^7^,^30^ – quantifies the relative change in steady state flux upon a change in enzyme concentration. For instance, 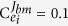 would mean that a 10% increase in *e*_*i*_ causes a 1% increase in *J*_*bm*_.

A relation therefore exists between the selection coefficient, the flux-control coefficient, and the enzyme concentration fraction. Intuitively, this result can be interpreted as follows: If 
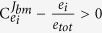
 an increase in the concentration of enzyme *i* by leads to a relative increase in the biosynthetic flux (the ‘benefit’; quantified by 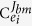) that outweighs the enzyme fraction (the ‘cost’, quantified by *e*_*i*_/*e*_*tot*_). Increasing the concentration of this enzyme will have a positive effect on fitness. Conversely, when 
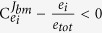
, reducing *e*_*i*_ increases fitness. Only when 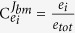 enzyme *i* is operating at its optimal concentration. The selection coefficient of a mutant with altered enzyme levels in the chemostat is therefore directly related to the distance of enzyme concentrations from optimality, defined by 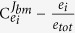. This relation therefore describes how changes in enzyme biochemistry and the topology of metabolic networks affect fitness in chemostats. In principle, one can find the optimal enzyme concentrations by solving 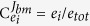 for all enzymes. However, in practice this is typically not the simplest way to do this.

A change in the concentration of an enzyme affects the concentrations of the limiting-nutrient (*s*, glucose in our model) and the metabolic product (*p*, ethanol). These dependencies turn out to be closely related to the selection coefficient, and are captured by the following relation (Supplementary Information S1),


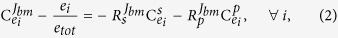


where 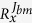 is the response coefficient of *J*_*bm*_ to *x*. It quantifies the relative change in *J*_*bm*_ in response to a change in the concentration of *x*, while keeping all other concentrations constant. 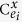 is the concentration control coefficient of enzyme *i* on substance *x*, quantifying the relative change in *x* when *e*_*i*_ is perturbed and a new steady state is reached^7^,^30^. This relation expresses the control of *e*_*i*_ on the cellular growth rate (left hand side of the equation) in terms of its effect on the extracellular nutrient concentrations that set the growth rate (right hand side of the equation). This indicates that the fitness of microorganism in the chemostat – the selection coefficient – is also determined by the current conditions in the chemostat.

**Figure 2:**
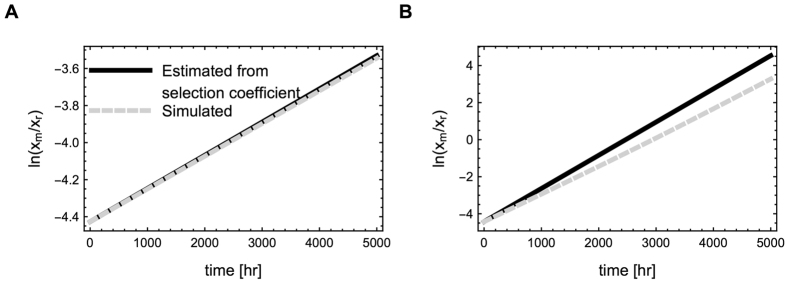
Selection coefficients estimated from flux control coefficients give a good estimate of the fixation rate of a mutant in the chemostat. This figure shows the logarithm of the biomass ratios of a resident and a mutant (black line). The mutant has a 1% (**A**) and 10% (**B**) reduced *e*_*ferm*_, but is otherwise equivalent to the resident. The selection coefficient, calculated from equation (1), can be used to predict the rate at which the mutant increases in abundance (gray dashed line). While this equation is technically only valid for infinitesimal changes in enzyme level, the result gives a good approximation of the rate at which a mutant takes over the population for more substantial changes in enzyme levels (the black and grey lines are very close in (**A**) and reasonably close in (**B**)).

Equation (1) allows us to estimate the rate at which a mutant can invade, given the flux control coefficient and the enzyme concentration of the enzyme that changes expression due to the mutation. To illustrate this, we calculated the steady state of a chemostat with a single respiro-fermentative strain present, the ‘resident’, for which we used arbitrarily chosen enzyme concentrations. The control coefficients are calculated numerically by changing the enzyme level slightly and recalculating intracellular steady state fluxes, while keeping the external glucose and ethanol concentration constant. For this strain, a decrease in the fermentation enzyme level, *e*_*ferm*_, had the largest effect on the fitness, with 
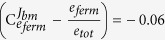
. We predicted the rate at which a mutant with a 1% reduction in *e*_*ferm*_ takes over the chemostat and confirmed this prediction with a simulation of the chemostat dynamics (Fig. 2A). Equation (1) is only strictly true for infinitesimal changes in enzyme levels. Since larger changes might occur due to mutations or experimental modification, we also test if the selection coefficient is a good approximation of the rate at which a mutant might take over the chemostat for larger changes in enzyme levels. For this, we predicted the rate at which a mutant with a 10% reduction in *e*_*ferm*_ would takes over the chemostat and compared it with a simulation (Fig. 2B). The agreement between the estimate and the simulation is reasonable, indicating that Equation (1) can give a good approximation even in the case of significant changes in enzyme concentrations.

The concentration of an enzyme is optimal when neither its increase nor its decrease enhances the selection coefficient (fitness). In other words, the selection coefficient of a mutant with an infinitesimal change in enzyme concentration must be zero. For an optimal phenotype, the following must therefore hold for each enzyme:


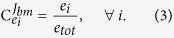


This is the same condition for optimality as found by Berkhout *et al.*^11^ for growth in mid-exponential batch conditions (at nutrient excess). This result was previously derived by Klipp and Heinrich^31^, albeit in a different context. Furthermore, combining equations 2 and 3 shows that in the optimum the residual glucose concentration is minimized and that the total biomass concentrations is maximized (Supplementary Information S1).

**Figure 3:**
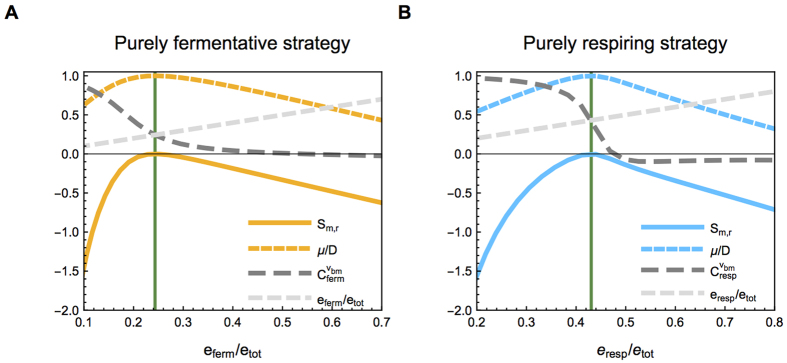
Fitness is maximal when all flux control coefficients are equal to the corresponding relative enzyme concentrations. The selection coefficient (solid colored lines), growth rate normalized to the dilution rate (dashed colored lines), control coefficient (dashed dark gray line), and relative enzyme concentration (dashed light gray lines) are shown as a function of the relative concentration of (**A**) fermentation enzyme of a pure fermenter and (**B**) respiration enzymes of a pure respirer. First, the optimal distribution of all enzyme concentrations, and the resulting steady state extracellular glucose concentration, was calculated for both a purely fermenting and a purely respiring strain. The optimal fermentation (**A**) and respiration (**B**) enzyme concentration are indicated by the vertical green line. To assess the fitness of mutants with alternative enzyme concentration distributions, we vary the fermentation and respiratory enzyme concentration, while keeping the external glucose concentration fixed. The selection coefficient is negative for all enzyme concentrations, so no mutants with the same metabolic mode, but other enzyme concentration distributions, can invade. Indeed, in the optimum the flux control coefficient of the fermentation (respiration) enzyme is equal to the relative fermentation (respiration) enzyme concentration.

Equation 3 characterizes the optimal levels of protein expression by a fermenter and respirer at a given dilution rate and glucose feed concentration. Figure 3 shows the selection coefficient and growth rate of a fermenter (A) and a respirer (B), as a function of the concentration of fermentation and respiration enzymes, respectively. (For details on how to find the optimal state in a chemostat, see Supplementary Information S2.4) The growth rate has a maximum, equal to the dilution rate, when *S*_*m*,*r*_ = 0, indicating that indeed when equation 3 holds, no mutant employing the same strategy (but at different enzyme level) can invade.

### Invasion by alternative strategies shows evolutionary instability of single strategies

**Figure 4:**
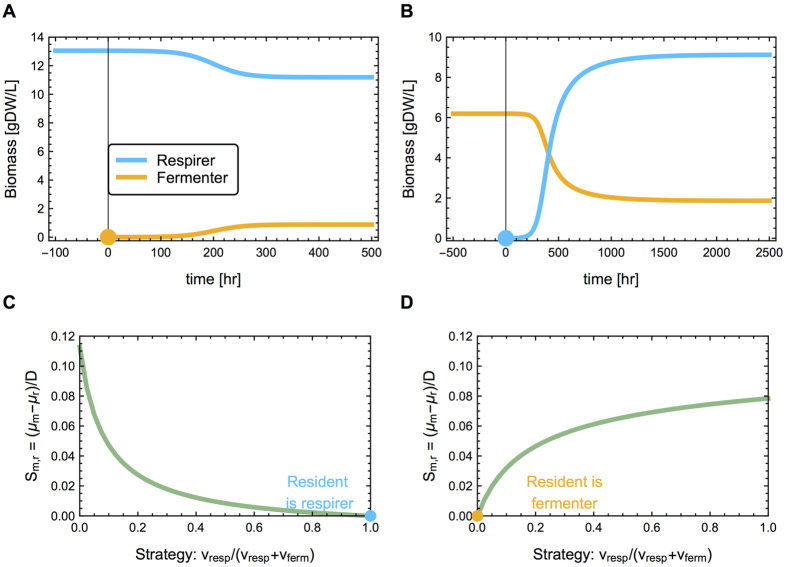
Respiring cells can invade a chemostat with an optimal, fermenter resident, and vice versa. The resident is optimal for the starting conditions of the chemostat (*D* = 0.3 hr^−1^ and *glu*_*feed*_ = 111 mM). This means that no other respiring strain can invade the chemostat in (**A**) and similarly no other fermenter in (**B**). (**A**,**B**) At *t* = 0, a ‘mutant’ of opposite metabolic strategy appears that settles in the chemostat, but does not take over completely. This indicates that (under these conditions) there is not a single optimal strategy in this chemostat. (**C**,**D**) The potential of opposing strategies to invade is clear from the fitness landscapes, which show the difference in growth rate, relative to the dilution rate, as a function of the relative flux through respiration. When a respirer (fermenter) is the resident, more fermenting (respiring) strategies have a positive selection coefficient, indicating that they have the potential to invade.

It might be tempting to conclude from the argumentation above that an evolutionarily stable situation must be a *single* pure strategy—in this case, a single genotype that either purely ferments or respires. However, for both the optimal fermenting and respiring strategy, the population can be invaded by a mutant employing the alternative strategy (Fig. 4A,B). These invaders will not take over completely, but a coexistence of two strains emerges. Because we start with strains that have optimal enzyme levels at the initial conditions (that is the steady state with only the optimal respirer (Fig. 4A) or only the optimal fermenter (Fig. 4B)), the respirer (and fermentor) strains in Fig. 4A,B do not have exactly the same enzyme levels and the biomass concentrations in the final state of coexistence differ.

The origin of this mutual potential for invasion can be illustrated by fitness-landscapes, defined as the dependency of the selection coefficient on the metabolic strategy. Figure 4C,D depict the selection coefficients of mutants in a resident population of respiring (4C) or fermenting (4D) cells. A phenotype with a positive selection coefficient can invade, but the mutant will affect the conditions in the chemostat, and therefore change the shape of the fitness landscape. For instance, when a fermenter invades, the concentration of ethanol in the vessel will increase, reducing the fitness advantage of the fermenting strategy until the ethanol concentration is such that the growth rate of the fermenters is equal to the dilution rate and a new steady state is reached. In conclusion, due to negative frequency-dependent selection, a coexistence between two phenotypes is possible.

**Figure 5:**
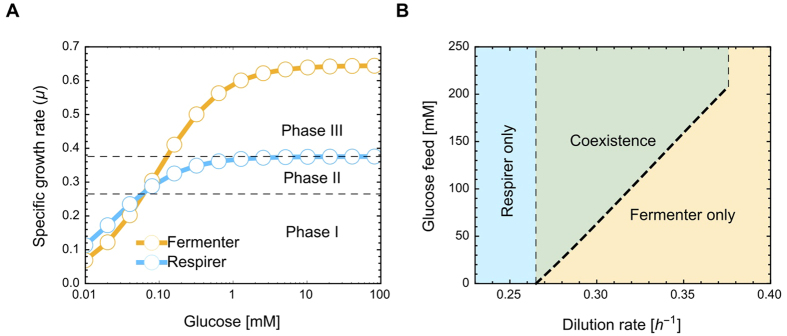
Requirements for coexistence. (**A**) The optimal specific growth rate of a respiring and a fermenting strain as a function of glucose concentration, in the absence of ethanol. At each glucose concentration, the optimal enzyme levels are calculated. There is a potential for coexistence in a chemostat when the dilution rate is such that in the absence of ethanol the fermenting strains outgrow the respiring strains, but the respirers will not wash out (Phase II). An additional requirement for coexistence is that the glucose concentration in the feed allows for enough accumulation of ethanol to inhibit the growth rate of fermenting cells to the extent that it equals the growth rate of respiring cells. Panel (**B**) shows combinations of glucose feed and dilution rate where coexistence can occur. We refer to the Supplementary Information S2.4 for details on how the borders between the phases (the dashed lines) can be calculated.

Whether in our model coexistence of a purely fermenting and a purely respiring strain is evolutionarily stable depends on the dilution rate and the glucose concentration in the feed. This dependency can be visualized by considering the growth rate of the (optimized) strains as a function of glucose concentration, in the absence of ethanol (Fig. 5A). We interpret the substrate concentration at which the cells achieve half of their maximal growth rate as the Monod constant. Figure 5 illustrates the dependency of this constant and the maximal growth rate on the metabolic strategy. Respirers have a lower Monod constant, whereas fermenters attain a higher maximal growth rate. Coexistence is impossible at low growth rates (phase I), because even in the absence of ethanol, respiring strains require a lower glucose concentration to attain a particular growth rate. At high growth rates (phase III), coexistence cannot occur because respiring strains will wash out. Phase II –with growth rates below the *μ*_*max*_ of the respiring strain, but where in the absence of ethanol, the fermenting strains will out-compete them– has the potential for stable coexistence. Fermenting cells initially outgrow respiring cells, which will lead to the accumulation of ethanol, inhibiting the growth of fermentative cells. When the glucose concentration in the feed is low, resulting in a low biomass concentration, ethanol will not accumulate to levels high enough to substantially inhibit the growth of the fermenting cells and a single fermenting strategy will be optimal. At higher feed concentrations ethanol will start to inhibit the fermenting cells and a stable coexistence will arise (Fig. 5B). The calculations of the dilution rates with stable coexistence can be found in Supplementary Information S2.4.

### Evolutionary dynamics: Diversification in the chemostat

We have shown that under some conditions any single strategy can be invaded by another strategy, which allows for diversification in a chemostat. To test this, we simulated the evolutionary dynamics in a chemostat, initialized with a resident employing a mixed, respiro-fermentative strategy. Our simulation methodology is similar to the method employed by Beardmore *et al.*^36^ and technical details can be found in the Supplementary Information S2.5. We divided the population into discrete genotypes, ranging from purely fermentative through respiro-fermenters to purely respiratory, each having different fractions of transporter protein. We vary both metabolic strategy and nutrient transport because of the focus on nutrient transport in the literature discussing evolution and selection pressures in the chemostat. Each genotype is defined by the relative flux to ethanol and respiration and the relative transport levels, but at each moment all enzyme levels are optimized for the prevailing conditions. The enzyme levels are allowed to take any value within the constraints of the genotype. This reflects the assumption that additional regulatory mechanisms regulate other aspects of the cellular resource allocation in an optimal manner. With these genotypes we can distinguish between selection on both metabolic mode and the transport protein. The chance that one genotype mutates into another decreases exponentially with the distance between the genotypes.

**Figure 6:**
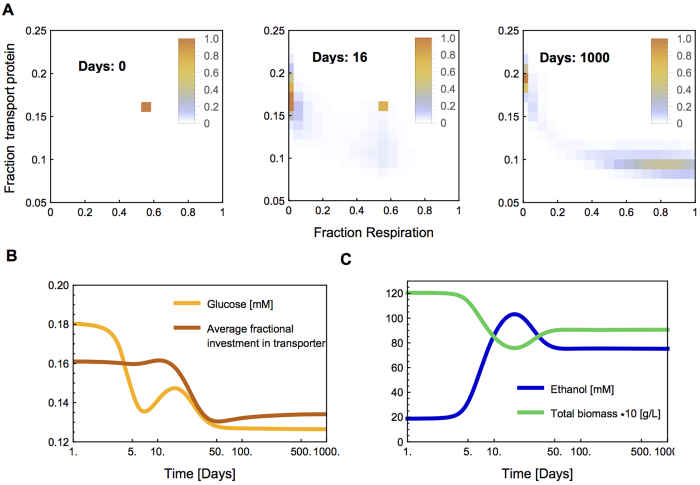
Diversification during simulated evolution in a chemostat environment. (**A**) The top panels show the time evolution of the biomass density of the different phenotypes. These phenotypes differ in their respiratory ratio and investment in transport proteins. First, fermenters starts invading, later followed by respirers. (**B**) The lower panels show the conditions in the chemostat and the average investment in transport protein during the first 1000 days of the simulated experiment, after that these are fairly constant. Note the logarithmic scale of the time axes.

We start the simulation with a resident employing a mixed, respiro-fermentative strategy and a transport protein fraction of about 16%. This population gradually evolves into different strategies, which also express different levels of transporters (Fig. 6 and Supplementary Video File). First, increasingly more fermentative mutants take over the population. As fermenters accumulate, so will the ethanol concentration in the chemostat, giving respiring strains a growth advantage. After some time, respirers indeed increase in numbers. In the end, the initial respiro-fermenters are completely replaced and a coexistence between respirers and fermenters results. The fermenters express more transporters than the initial respiro-fermenters, while the respirers express less, illustrating the complex interplay between metabolic strategy, optimal enzyme concentrations and growth characteristics. Optimal enzyme allocation can also lead to an decrease in the average investment of transport protein in the population (Fig. 6B).

There appear to exist three periods in which the biomass composition changes, as can be seen in the time evolution of the extracellular conditions and in the population changes (Fig. 6B,C) and the complete simulation in the Supplementary Video File. Between day 4 and day 15, the fermenters appear and settle. Because these fermenters have a lower biomass yield, this leads to a reduction in the total biomass. An intricate interplay between the abundances of the different phenotypes and the accumulating ethanol concentration during and after this period causes the residual glucose concentration to increase. As a consequence, between days 15 and 50 a respiro-fermenter with reduced transporter expression emerges, which become progressively more respiratory until fermentation stops between days 100 and 1000. This coincides with a decrease in average investment in transporter, indicating that selection in the chemostat does not always increase transporter levels (and neither does the affinity of the cell for the substrate always increase, see Supplementary Figure S3). The maintenance of several genotypes close to the optimal strategy in the final state—which might seem contradictory with our arguments above—is the result of a mutation-selection balance. At lower mutation rates, the total biomass is distributed over less genotypes in the evolutionarily stable state (data not shown). This simulation illustrates the potential for diversification in a homogeneous environment by way of negative frequency-dependent selection. Despite the complex and unpredictable dynamics during evolution, the evolutionarily stable endpoint can be predicted.

## Discussion

In this work we showed the theoretical possibilities for the evolution of metabolism in the chemostat; these are only single EFM strategies with a possibility for coexistence. We obtained a better understanding of how evolutionary pressures affect metabolic strategies and concentrations of metabolic enzymes. We took both the benefits and costs of protein expression into account and expressed the cellular growth rate in terms of the rate with which metabolism makes new biomass per unit biomass. In this manner, we could couple the extracellular dynamics of nutrients and inhibitors in the chemostat to the metabolic state and strategy of a growing cell. We found that selection acts in chemostat cultures in a similar fashion as it does under constant conditions^29^: The optimal metabolic strategy is an EFM that maximizes the growth rate at the prevailing conditions. However, the difference is that in the chemostat a coexistence of ‘pure’ EFM strategies can evolve. This result is unique for environments where the strains influence their environments, and would not hold if the environment is constant (e.g. a microfluidics setup with low glucose concentrations where the products are directly washed away), because the negative feedback through the environment is essential for the coexistence. We derived theory that predicts the conditions for the optimal concentrations of enzymes, nutrients and inhibitors in the chemostat. We illustrated how species diversification can occur in a homogeneous and apparently (but not quite) constant environment, using a coarse-grained self-replicator model of *S. cerevisiae*. The fact that the fermentation product inhibits the fermenting strain effectively creates a new niche, as it allows the fermenter to grow equally fast as the respirer, leading to coexistence. An alternative mechanism for niche creation could be a cross-feeding intermediate, which we did not consider in our modeling efforts for the sake of simplicity and clarity.

In our evolutionary simulation an equilibrium was reached, evolutionary cycles were not observed. However, we cannot exclude that such cycles are possible.

We show that the Monod constant—quantifying the affinity of microorganism for the limiting substrate^37^—does not necessarily have to decrease during prolonged cultivation in the chemostat. The Monod constant typically depends on the expression of all proteins and is therefore not, as is often suggested, necessarily equal to the half-saturation constant of the transporter of the limiting substrate^12^,^28^.

Our results indicate that in the chemostat environment coexistence of two or more different metabolic strategies can be an evolutionarily stable state. Classically, in game-theory, a population of individuals employing a mixed strategy is equivalent to a coexistence of two populations employing each a pure strategy^38^. A mixed metabolic strategy, however, is not equivalent to a coexistence in our case, because a mixed strategy is not an EFM. The difference arises from the fact that the pure EFMs occur in different organisms and therefore operate at different intracellular concentrations in the respiring and fermenting cells. For instance, a fermenting cell benefits from a high intracellular glucose concentration, because the affinity of fermentation for intracellular glucose is low. The respiration metabolic-mode has a high affinity for intracellular glucose and respiring cells benefit from a low level of intracellular pyruvate, because it decreases the product inhibition on glycolysis. A cell employing a mixed strategy must compromise between these two, and as a consequence is less fit than the pure strategies. Even though mixed strategies are not optimal, the different pure strategies do not necessarily have to be genetically hardwired. The subpopulations can also arise from phenotypic plasticity, as long as each subpopulation exhibits a pure strategy.

Coexistence of different phenotypes in the chemostat has often been observed^39^,^40^,^41^,^42^,^43^,^44^, and several theoretical explanations have been suggested. Phenomenological models of microbial growth show multiple mechanisms that can lead to stable coexistence: cross-feeding^45^; negative frequency-dependent selection through inhibition–either by a waste product^46^ or by an antibiotic substance^47^; or a combination of a rate-affinity and rate-yield trade-off^36^,^48^. These models do not take metabolism and protein costs explicitly into account and therefore have to postulate the fitness effects leading to coexistence. Pfeiffer and Bonhoeffer showed that under particular conditions coexistence of cross-feeders and (partial or complete) glucose degraders can be an evolutionarily stable state, due to inhibition by – and feeding on – a glycolytic ‘waste’ product^49^. While they did take enzyme costs into account, they simply postulated them to be present, but did not derive them. Furthermore, they required particular assumptions about the toxicity of metabolic intermediates, and modeled growth rate indirectly. Our analysis adds to these works that we identify ‘pure’ metabolic strategies (i.e. EFMs) as the only evolutionarily stable strategies, we derive the effects of enzyme costs directly from a model, which integrates metabolism and growth, and we show explicitly that coexistence can evolve from a single ancestor, regardless of its starting phenotype.

In our models ethanol cannot be consumed. This can however be added to our modelling framework. This would open up the possibility for coexistence of crossfeeding, for which the conditions can be determined in a similar fashion as done in this article. A survey of this setting was done by Pfeiffer *et al.*^49^, albeit not in a fully mechanistic model. The results of the same analysis with the framework proposed in this article is expected to give qualitatively similar results.

Several researchers have evolved *S. cerevisiae* and *E. coli*^39^ in the chemostat (summarized in Gresham & Hong^50^). These experiments have been conducted at low dilution rates, possibly for practical reasons, such as medium supply. These dilutions rates are below the threshold for coexistence in our analysis, and, indeed, a shift from mixed to fully respiratory metabolism and an increased yield were observed in these evolution experiments^51^,^52^,^53^. Prolonged evolution in a chemostat often leads to increased substrate transport capacity, which is associated with an increased limiting-substrate affinity of the cells, and hence their fitness^50^. The most straightforward manner for a cell to enhance its transport capacity is however through increased expression of the transporter proteins, illustrating the importance of the adaptation of protein levels to the environment for fitness.

The stability of species coexistence due to inhibitory compounds has been shown experimentally with a coexistence of *S. cerevisiae* and *E. coli*^44^ strains. This study also indicated that coexistence can depend on the feed concentration, in agreement with our predictions. In principle those experiments can be repeated with two *S. cerevisiae* strains that differ in their respiratory ratio. These strains can be natural strains^33^,^54^ or modified strains. For the coexistence to be stable it is required that the fermenting strains should grow faster than the respiring strain, at a certain dilution rate, and that the fermenting strain should be inhibited enough by ethanol (to make coexistence possible).

When comparing theory with experimental results, it has to be kept in mind that microorganisms are likely not optimized for the chemostat environment, nor can we expect that cells are optimal after prolonged evolutionary experiments. In addition, selective pressures can act on other processes than the allocation of biosynthetic resources. For instance, limitations in membrane area could help explain the evolution of cell size and shape^28^ and the catalytic properties of enzymes might also be subject to selection^55^,^56^. An understanding of the intricate interplay of selective pressures on different aspects of cellular functioning, as well as an understanding of which pressures are dominant under particular conditions, requires first a proper understanding of how selection acts on each aspect individually.

## Additional Information

**How to cite this article**: Wortel, M. T. *et al.* Evolutionary pressures on microbial metabolic strategies in the chemostat. *Sci. Rep.* **6**, 29503; doi: 10.1038/srep29503 (2016).

## Supplementary Material

Supplementary Video File

Supplementary Information
