# Supplementary material for: Evolutionary pressures on microbial metabolic strategies in the chemostat
Source: Sci Rep. 2016 Jul 6;6:29503. doi: 10.1038/srep29503 (PMC4933952; doi:10.1038/srep29503)
Supplement: Supplementary Information [file srep29503-s2.pdf]

Supplementary Information to: Evolutionary pressures on microbial metabolic strategies in the chemostat

Meike T Wortel[1, 2, +], Evert Bosdriesz[1, 3, +], Bas Teusink[1], and Frank J Bruggeman[1, *]

[1]Systems Bioinformatics, VU University, Amsterdam, De Boelelaan 1087, 1081 HV, The Netherlands
[2]Present address: Centre for Ecological and Evolutionary Synthesis (CEES), the Department of Biosciences, University of Oslo, Blindernveien 31, 0371 Oslo, Norway
[3]Present address: Division of Molecular Carcinogenesis, The Netherlands Cancer Institute, Plesmanlaan 121, 1066 CX Amsterdam, The Netherlands
[*]f.j.bruggeman@vu.nl
[+]These authors contributed equally

S1 Derivations

S1.1 Selection coefficient of a mutant in a chemostat

Here, we will derive an expression that relates the selection coefficient to the flux control coefficient and enzyme concentration. To this end, we will consider what happens when at $t = 0$ a mutant m arises in a population of resident cells, r, that were initially in a chemostat at steady state. Because we are interested in how enzyme concentrations affect fitness, we assume that mutation only affects metabolic enzyme concentrations and not their kinetics parameters, such as k_{cat}s or K_Ms.

The selection coefficient between two strains m and r, $\mathscr{S}_{m,r}$, is defined as the time-derivative of logarithm of the frequencies of m and r.[1] For convenience, it can be normalized to the dilution rate of the chemostat, D.

$$\begin{aligned}\mathscr{S}_{m,r} &= \frac{1}{D}\frac{\mathrm{d}}{\mathrm{d}t}\ln\frac{m}{r} \\ &= \frac{1}{D}\left(\frac{1}{m}\frac{\mathrm{d}m}{\mathrm{d}t} - \frac{1}{r}\frac{\mathrm{d}r}{\mathrm{d}t}\right) \qquad \text{(S1)} \\ &= \frac{1}{D}(\mu_m - \mu_r), \qquad \text{(S2)}\end{aligned}$$

where we've used that $\frac{1}{m}\frac{\mathrm{d}m}{\mathrm{d}t} \equiv \mu_m$, and equivalently for μ_r. Note that $\mathscr{S}_{m,r}$ is not necessarily constant in time; it becomes time dependent when the number of mutant organisms becomes of the order of the number of resident organisms. We consider the situation when this has not yet occurred. Generally, the growth rate of a cell in the chemostat is a function of its enzyme concentrations $\mathbf{e}$, the residual substrate concentration s and, potentially, on the concentration of the waste product, p, i.e. $\mu = \mu(\mathbf{e}, s, p)$. The resident has enzyme concentrations $\mathbf{e}_r$, and we denote the substrate and product concentration at $t \leq 0$ with s_0 and p_0, respectively. Before the mutation arises it thus holds that $\mu_{r,0} \equiv \mu(\mathbf{e_r}, s_0, p_0) = D$. Suppose that the mutation only affects enzyme i, such that the mutant has $e_{i,m} = e_{i,r} + \delta e_i$, but is otherwise equivalent to the resident. This will affect the growth rate of m, but also that of r through its effect on the residual substrate concentration s and, potentially, on waste product concentration p. If we assume that the effect of the mutation is small, we can use a first order Taylor-expansion around $\mu_{r,0}$ to approximate μ_r and μ_m, the growth rates of the resident and the mutant after the mutations arose:

$$\mu_r \approx \mu_{r,0} + \left.\frac{\partial\mu(\mathbf{e},s,p)}{\partial s}\right|_{\{\mathbf{e}_r,s_0,p_0\}} \delta s + \left.\frac{\partial\mu(\mathbf{e},s,p)}{\partial p}\right|_{\{\mathbf{e}_r,s_0,p_0\}} \delta p \qquad \text{(S3a)}$$

$$\mu_m \approx \mu_{r,0} + \left.\frac{\partial\mu(\mathbf{e},s,p)}{\partial s}\right|_{\{\mathbf{e}_r,s_0,p_0\}} \delta s + \left.\frac{\partial\mu(\mathbf{e},s,p)}{\partial p}\right|_{\{\mathbf{e}_r,s_0,p_0\}} \delta p + \left.\frac{\partial\mu(\mathbf{e},s,p)}{\partial e_i}\right|_{\{\mathbf{e}_r,s_0,p_0\}} \delta e_i \qquad \text{(S3b)}$$

Note that the terms δs and δp are time-dependent, but initially, when the mutant just appears, they are equal to zero, since the mutant has not affected the chemostat yet. Equation (S3) is valid with $\delta s = 0$ and $\delta p = 0$ during this early stage. Then, all

terms cancel, except the last; such that:

$$\mathscr{S}_{m,r} = \frac{e_i}{D}\frac{\partial \mu}{\partial e_i}\frac{\delta e_i}{e_i} \tag{S4}$$

By definition, the specific growth rate is the rate of biomass synthesis, J_{bm}, per unit biomass,

$$\mu \equiv \frac{J_{bm}}{e_{tot}}, \tag{S5}$$

where e_{tot} is the total enzyme content of the cell and we ignore other biomass components. Combing equation S4 and S5, we arrive at:

$$\mathscr{S}_{m,r} = \frac{1}{D}\left(\frac{e_i}{e_{tot}}\frac{\partial J_{bm}}{\partial e_i} - \frac{e_i J_{bm}}{e_{tot}^2}\right)\frac{\delta e_i}{e_i} \tag{S6}$$

$$= \left(\mathrm{C}_{e_i}^{J_{bm}} - \frac{e_i}{e_{tot}}\right)\frac{\delta e_i}{e_i} \tag{S7}$$

This remains true during the potential wash-in of mutant m. In the second step we used that $\mu = D$.

This result can straightforwardly be generalized to a mutation that affects several (or all) enzyme concentrations; $\mathbf{e} \to \mathbf{e} + \delta \mathbf{e}$. In that case:

$$\mathscr{S}_{m,r} = \sum_i \left(\mathrm{C}_{e_i}^{J_{bm}} - \frac{e_i}{e_{tot}}\right)\frac{\delta e_i}{e_i} \tag{S8}$$

S1.1.1 Initial selection coefficient of a mutant against a coexisting strain

When the initial steady state in the chemostat is a coexistence of two species, r_1 and r_2, one might also be interested in the selection coefficient of a mutant m_1, stemming from r_1, against r_2. We know that prior to r_1 arising, μ_{r_1} and μ_{r_2} must be equal to μ_0. However, since $\frac{\partial \mu_{r_1}}{\partial s}$ and $\frac{\partial \mu_{r_2}}{\partial s}$ are not necessarily equal, these terms do not cancel in the same way as in equation (S3). However, initially $\delta s = 0$ and $\delta p = 0$ and hence equation (S8) initially is a good approximation of the selection coefficient of mutant m_1 against resident r_2.

S1.2 The relation between flux and concentration control coefficients

Consider what happens when we perturb the level of enzyme i, e_i, of all cells in a chemostat. This will have some immediate effect on the growth rate of the organism, which will affect the residual substrate concentration, s, and potentially the product concentration p. Eventually, the system will reach a new steady state. However, the design of a chemostat is such that the growth rate in the initial and final steady state are equal. This implies the following equality:

$$0 = \frac{\mathrm{d}\mu}{\mathrm{d}e_i} = \frac{\partial \mu}{\partial e_i} + \frac{\partial \mu}{\partial s}\frac{\mathrm{d}s}{\mathrm{d}e_i} + \frac{\partial \mu}{\partial p}\frac{\mathrm{d}p}{\mathrm{d}e_i} \tag{S9}$$

where $\frac{\mathrm{d}x}{\mathrm{d}e_i}$ denotes the change in the concentration of x in response to a change in e_i *when the system has reached its new steady state*. Substituting $\mu = J_{bm}/e_{tot}$, taking the derivatives and reordering terms gives:

$$\frac{1}{e_{tot}}\frac{\partial J_{bm}}{\partial e_i} - \frac{J_{bm}}{e_{tot}^2} = -\frac{1}{e_{tot}}\frac{\partial J_{bm}}{\partial s}\frac{\mathrm{d}s}{\mathrm{d}e_i} - \frac{1}{e_{tot}}\frac{\partial J_{bm}}{\partial p}\frac{\mathrm{d}p}{\mathrm{d}e_i}.$$

Multiplying both sides by $e_i e_{tot}/J_{bm}$, this is equivalent to:

$$\mathrm{C}_{e_i}^{J_{bm}} - \frac{e_i}{e_{tot}} = -R_s^{J_{bm}}\mathrm{C}_{e_i}^{s} - R_p^{J_{bm}}\mathrm{C}_{e_i}^{p} \tag{S10}$$

where $R_s^{J_{bm}} \equiv \frac{s}{J_{bm}}\frac{\partial J_{bm}}{\partial s}$ and $R_p^{J_{bm}} \equiv \frac{p}{J_{bm}}\frac{\partial J_{bm}}{\partial p}$ are the local response coefficients of the biomass synthesis flux to a change in the concentration of s and p, respectively. Since s is a limiting substrate, $R_s^{J_{bm}}$ is positive and since p is an inhibitor, $R_p^{J_{bm}}$ is negative (or zero for cells not sensitive to p). Furthermore $\mathrm{C}_{e_i}^{s} \equiv \frac{e_i}{s}\frac{\mathrm{d}s}{\mathrm{d}e_i}$ and $\mathrm{C}_{e_i}^{p} \equiv \frac{e_i}{p}\frac{\mathrm{d}p}{\mathrm{d}e_i}$ are the concentration control coefficient of e_i on s and p, respectively. These are the scaled change in residual substrate or product concentration in response to a change in the enzyme concentration, after the chemostat reaches its new steady state.

In the main text we show that in the optimum $C^{J_{bm}}_{e_i} = \frac{e_i}{e_{tot}} \forall i$. Combining this with equation S10 we find that in the optimum,

$$R^{J_{bm}}_s C^s_{e_i} = -R^{J_{bm}}_p C^p_{e_i}, \quad \forall i. \tag{S11}$$

$R^{J_{bm}}_s$ and $R^{J_{bm}}_p$ always have an opposite sign, as s and p are the substrate and the product of the metabolic system, respectively. In addition, for an EFM, $C^s_{e_i}$ and $C^p_{e_i}$ have opposite signs, and therefore the LHS and RHS of equation (S11) have opposite signs. Since $R^{J_{bm}}_s$ and $R^{J_{bm}}_p$ can never both be strictly zero, this implies that the optimal strategy is characterized by $C^s_{e_i} = 0$ and $C^p_{e_i} = 0$, $\forall i$, i.e. the substrate and product concentration in the chemostat both reach either an extremal value when the enzyme concentrations are optimal. Logically, the substrate concentration will have a minimum and the product concentration a maximum. This has a consequence for the biomass concentration in the chemostat. Since EFMs have fixed, net-reaction stoichiometries, in other words all the product-over-substrate yield of an EFM are fixed, and, since the substrate concentration reaches a minimum in the optimum, the biomass concentration in the chemostat is maximized in the optimum.

S2 Model description

In order to illustrate our theoretical results and show what implications they might have, we have constructed a model of *S. cerevisiae* in a chemostat environment. Our model can be divided in two parts: One describing the metabolism and growth of the *S. cerevisiae* cells, and one describing the dynamics of biomass, nutrients and waste-products in the chemostat. We assume that the timescales of metabolism are much faster than the dilution rate of the vessel. This allows us to model these two parts separately.

In formulating our model of *S. cerevisiae* growth, we will ensure the model has the following properties:

- At low glucose concentrations respiration is more efficient than fermentation, whereas at high glucose concentrations fermentation is more efficient. As a result, the optimal metabolic strategy is condition dependent.
- Fermentation is inhibited by ethanol, but respiration is not. As a results, negative frequency depend selection against fermenting strategies can arise.

These properties are biologically plausible, but not necessarily true. We will use literature data and model fitting to ensure our model is operating under realistic conditions. However, it is explicitly not our intention to provide an as realistic as possible model of *S. cerevisiae*. Rather, we use it to illustrate how such properties give rise to certain peculiar evolutionary dynamics and how mathematical insights help in understanding and analyzing these.

S2.1 Self replicator model of *Saccharomyces Cerevisiae*

Our model of *S. cerevisiae* metabolism and growth is based on the concept of self-replicator models as introduced by.[2] The model is named self-replicator because although relatively simple, it is a self contained representation of cellular replication. It contains modules that can represent single enzymes, enzyme complexes or whole pathways: namely glucose transport proteins (HXT), glycolysis (gly), ethanol production (ferm), respiration including mitochondria (resp) and ribosomes (rib) (Figure 1 in the main text). The reaction rates of the modules are modeled by saturation kinetics (Michaelis-Menten kinetics). Since different strains of *S. cerevisiae* have slightly different enzymes with different kinetic properties, we have made a model of an "average" *S. cerevisiae* cell, taking the best measurements or the averages, in case of more than one valid measurement, of the data we obtained from literature.

The dynamics of the intracellular metabolites are described by ordinary differential equations (ODEs). We assume that the total amount of ADP and ATP is constant. With this moiety conservation we replace ADP by a AXP - ATP and only keep track of the changes in ATP. Therefore the model consists of the following ODE's:

$$\frac{d\mathrm{glu}_i}{dt} = v_{\mathrm{hxt}} - v_{\mathrm{gly}} \tag{S12a}$$

$$\frac{d\mathrm{pyr}}{dt} = 2v_{\mathrm{gly}} - v_{\mathrm{rib}} - v_{\mathrm{ferm}} - v_{\mathrm{resp}} \tag{S12b}$$

$$\frac{d\mathrm{ATP}}{dt} = 2v_{\mathrm{gly}} + 9v_{\mathrm{resp}} - 2.4v_{\mathrm{rib}} \tag{S12c}$$

where glu_i, pyr and ATP represents the intracellular glucose, pyruvate and ATP concentration, respectively.

The stoichiometry coefficients were calculated with data from literature. Protein assembly costs 4 ATP per amino acid.[3] There is an average of 5 C-atoms per amino acid, and pyruvate consists of 3 C-atoms. Hence, per pyruvate used in biomass synthesis, 2.4 ATPs are used. For respiration we use 9 ATP per pyruvate, derived from the maximum of 18 ATP per glucose from respiration.[4]

We describe the model with module rate equations, which resemble Michaelis-Menten kinetics with product inhibition. The module-k_{cat}s are interpreted as the turnover number of the whole module, in the unit mol substrate per mol C-atom in the module per hour. Because the module-k_{cat}s are per mol C-atom in the module, larger modules will have lower module-k_{cat}s. Alternatively we could calculate the k_{cat}s per module and apply weights to the pathways according to their size (e.g. the number of C-atoms), because larger modules are more expensive to make. This second option would lead to the exact same result, but we chose to reflect the pathway costs in the module-k_{cat}s.

Parameter	Value [mM]	Reference
$K_{M,\text{glu,hxt}}$	1.19	[5]
$k_{i,\text{hxt}}$	0.91	[5]
$K_{M,\text{glu}_i,\text{gly}}$	0.08	[5]
$K_{M,\text{ADP,gly}}$	1	arbitrary
$K_{P,\text{pyr,gly}}$	21	[5]
$K_{M,\text{pyr,ferm}}$	6	[6]
$K_{P,\text{EtOH,ferm}}$	17	[5]
$K_{M,\text{pyr,resp}}$	0.13*	[7]
$K_{M,\text{ADP,resp}}$	1	arbitrary
$K_{M,\text{pyr,rib}}$	1	arbitrary
$K_{M,\text{ATP,rib}}$	1	arbitrary
AXP	3.1	[8]

Table S1. Kinetic parameters of the module reactions. *This value of the affinity for pdh was obtained at a more realistic pH of 6.5 (and comparable to the pH at which pdc was assayed)

The rate equations are given by:

$$v_{\text{hxt}} = k_{cat,\text{hxt}} \cdot e_{\text{hxt}} \frac{\frac{\text{glu}-\text{glu}_i}{K_{M,\text{glu,hxt}}}}{1+\frac{\text{glu}}{K_{M,\text{glu,hxt}}}+\frac{\text{glu}_i}{K_{M,\text{glu,hxt}}}+k_{i,\text{hxt}}\frac{\text{glu}\cdot\text{glu}_i}{K_{M,\text{glu,hxt}}K_{M,\text{glu,hxt}}}} \tag{S13a}$$

$$v_{\text{gly}} = k_{cat,\text{gly}} \cdot e_{\text{gly}} \frac{\frac{\text{glu}_i}{K_{M,\text{glu}_i,\text{gly}}}\frac{\text{ADP}}{K_{M,\text{ADP,gly}}}}{\left(\frac{\text{glu}_i}{K_{M,\text{glu}_i,\text{gly}}}+1\right)\left(\frac{\text{ADP}}{K_{M,\text{ADP,gly}}}+1\right)\left(1+\frac{\text{pyr}}{K_{P,\text{pyr,gly}}}\right)} \tag{S13b}$$

$$v_{\text{ferm}} = k_{cat,\text{ferm}} \cdot e_{\text{ferm}} \frac{\text{pyr}}{(K_{M,\text{pyr,ferm}}+\text{pyr})(1+\frac{\text{EtOH}}{K_{P,\text{EtOH,ferm}}})} \cdot \frac{1}{1+e^{h(\text{ATP}-K_{I,ATP})}} \tag{S13c}$$

$$v_{\text{resp}} = k_{cat,\text{resp}} \cdot e_{\text{resp}} \frac{\frac{\text{pyr}}{K_{M,\text{pyr,resp}}}\frac{\text{ADP}}{K_{M,\text{ADP,resp}}}}{\left(\frac{\text{pyr}}{K_{M,\text{pyr,resp}}}+1\right)\left(\frac{\text{ADP}}{K_{M,\text{ADP,resp}}}+1\right)} \cdot \frac{1}{1+e^{h(\text{ATP}-K_{I,ATP})}} \tag{S13d}$$

$$v_{\text{rib}} = k_{cat,\text{rib}} \cdot e_{\text{rib}} \frac{\frac{\text{pyr}}{K_{M,\text{pyr,rib}}}\frac{\text{ATP}}{K_{M,\text{ATP,rib}}}}{\left(\frac{\text{pyr}}{K_{M,\text{pyr,rib}}}+1\right)\left(\frac{\text{ATP}}{K_{M,\text{ATP,rib}}}+1\right)} \tag{S13e}$$

The parameter values for the rate equations are given in Table S1. The affinity of a pathway is mostly determined by the affinity of the first enzyme (Pınar Kahraman, personal communication), so where possible we took that affinity. The rate equations v_{resp} and v_{ferm} are multiplied by an extra factor $(1-e^{h(\text{ATP}-K_{I,ATP})})^{-1}$. This is done to make the model more stable. Otherwise, when e_{resp} or e_{ferm} are too high, these fluxes "outcompete" the ribosome for pyruvate, causing ATP to accumulate and ADP to deplete, which in turn inhibits glycolysis. As a result, the model tends to a "trivial" steady state where pyr $\to 0$, ATP $\to$ AXP and $\text{glu}_i \to \text{glu}$. We consider this an unrealistic model artifact. The extra factor $(1-e^{h(ATP-K_{I,ATP})})^{-1}$ is an ad-hoc way to solve this problem. We assume that the cell has mechanisms to avoid ADP and ATP from being depleted, but those mechanisms are outside the scope of our model. By setting $K_{I,ATP}$ close to, but below, AXP, and n_{ATP} high (we use $K_{I,ATP} = 2mM$ and $h = 5$) this term will normally be close to 1, and when ATP exceeds $K_{I,ATP}$, it will quickly tend to 0 with increasing ATP, preventing the model from entering the "trivial" steady state. This term does not influence the behavior of the model near optimal states.

The specific growth rate, μ, is defined as the biomass synthesis rate per unit biomass (for details, see[2]). In our model, biomass only consists of enzymes, and enzyme concentrations are in mmol C-atoms. Since v_{rib} is in mM pyruvate consumed per hour, and pyruvate contains 3 C-atoms, we have to make a correction to the ribosome rate to obtain the biomass production rate (v_{bm}). Hence, the specific growth rate is given by

$$\mu = \frac{v_{bm}}{e_{\text{tot}}} \quad \text{with } v_{bm} = 3 \cdot v_{rib}. \tag{S14}$$

The rate of biomass synthesis depends on the external glucose and ethanol concentration, glu and EtOH, and on the relative enzyme concentrations. Note that μ does not depend on the absolute total enzyme concentration. For instance, if all enzyme

concentrations are doubled, all rates will double as well, canceling each other in equation (S14). We can thus calculate the maximal growth rate (for given glu and EtOH) by either fixing e_{tot} and maximizing v_{rib}, or fixing v_{rib} and minimizing e_{tot}. The latter is often computationally more efficient.

S2.2 Chemostat model

Since we assume the timescales at which metabolism operates are much faster than the dilution rate, we can use the model above to calculate the growth rate of a cell (with given enzyme levels $\mathbf{e}$) in a chemostat, as a function of glu and EtOH, i.e. $\mu = \mu(\mathbf{e}, \text{glu}, \text{EtOH})$. The dynamics of biomass, glucose and ethanol in a chemostat are governed by the following set of ODEs:

$$\frac{\mathrm{d}X}{\mathrm{d}t} = (\mu - D) \cdot X \tag{S15a}$$

$$\frac{\mathrm{dglu}}{\mathrm{d}t} = D(\text{glu}_{feed} - glu) - \frac{1}{Y_{X/S}} \cdot \mu \cdot X \tag{S15b}$$

$$\frac{\mathrm{dEtOH}}{\mathrm{d}t} = \mu \cdot Y_{\text{EtOH}/X} \cdot X - D \cdot \text{EtOH} \tag{S15c}$$

where D is the dilution rate, X the biomass concentration, glu_{feed} the glucose concentration in the feed, $Y_{X/S} \equiv v_{\text{bms}}/v_{\text{hxt}} \cdot \gamma$ the yield of biomass on glucose in gram biomass per mmol glucose and $Y_{\text{EtOH}/X} \equiv v_{\text{ferm}}/v_{\text{bms}} \cdot 1/\gamma$ the amount of ethanol produced per unit biomass produced in mmol ethanol per gram biomass. γ is a conversion factor that captures the gram biomass per millimole of C-atoms in the biomass, which we calculated from the average of the protein data used to estimate the pathway costs (Section S2.3) to be 22.36 gram millimole^{-1}.

These equations are easily generalized to a situation where more than one species is present in the chemostat. For N species with different enzyme levels $\mathbf{e}_i$ and growth rates $\mu_i \equiv \mu(\mathbf{e}_i, \text{glu}, \text{EtOH})$, the dynamics of the chemostat are given by:

$$\frac{\mathrm{d}X_i}{\mathrm{d}t} = (\mu_i - D) \cdot X_i \qquad \forall i \in \{1, \ldots, N\} \tag{S16a}$$

$$\frac{\mathrm{dglu}}{\mathrm{d}t} = D(\text{glu}_{feed} - glu) - \sum_{i=1}^{N} \frac{1}{Y_{X/S,i}} \cdot \mu_i \cdot X_i \tag{S16b}$$

$$\frac{\mathrm{dEtOH}}{\mathrm{d}t} = \sum_{i=1}^{N} (\mu_i \cdot Y_{\text{EtOH}/X,i} \cdot X_i) - D \cdot \text{EtOH} \tag{S16c}$$

S2.3 Determination of the pathway costs and fitting to literature data

In formulating our model, we used literature values for the parameters as much as possible. When this was not possible, we established them by fitting to literature data. Below, details of these procedures are described, but the overall approach was to:

- Take into account the enzyme investment required for each module.
- Get a maximal growth rate consistent with literature data.
- Make respiration more efficient at low, and fermentation more efficient at high glucose concentration.

In total we fit two parameters to literature data, but these are used to "rescale" many of the model parameters.

The module k_{cat}s are defined as turnover per unit enzyme per hour. It is not possible to directly relate this to the k_{cat}s of the enzymes that constitute the module, and no experimental data of these is available either. However, some modules contain much more (for example the respiration module) or much larger (the ribosome) enzymes than the average module. Therefore, the relative k_{cat}s are estimated based on the number and sizes of the enzymes that make up the module, so as to account for these "module costs". To this end, we counted the C-atoms in the amino acid composition of the proteins in the modul (obtained from UniProt[9]). Table S2 lists UniProt ID's associated with each modules. There are 11984 amino acids in the small and large subunit of the ribosome.[10] The proteins from the other pathways had an average of 5-carbon atoms per amino acid, and assuming that this is the same for the ribosomal proteins, we have calculated the carbon content of a ribosome. This is the same order of magnitude as when we calculate this number from the weight of the subunits: $1.05 \cdot 10^6$ daltons for the large subunit and $0.57 \cdot 10^6$ daltons for the small subunit.[11] When we multiply by the number of C-atoms per dalton in the other pathways we obtain 72465 C-atoms.

The module costs allow us to establish the module k_{cat}s relative to one another, but not their absolute values. To determine these, we used literature data on the maximal specific growth rate. Since the maximal growth rate is reached by fully fermenting cells, we could fit the pathway turnover number to a realistic maximal growth rate. The maximal specific growth rate that we

Module	Cost in C-atoms	Uniprot IDs
Transport	2879.33	hxt (N1NXQ9; N1P133; N1P4M3; N1PAD0; N1P5B7; N1P268; N1P0E9; N1P9E5; N1P8P4)
Glycolysis	21225.17	hxk (P17709; P04806; P04807), pgi (P12709), pfk (P16861; P16862), ald (P14540), gapdh (P00359; P00360; P00358), pgk (P00560), pgm (Q12008; P00950; Q12326), eno (P00924; P00925), pyk (P00549; P52489)
Fermentation	4601.25	pdc (P06169; Q07471; P16467; P26263), adh (P38113; P32771; P10127; P07246; P00331; P00330; P25377; Q04894)
Respiration	???	*Fitted*
Ribosome	59920	*Calculated* see text

Table S2. Proteins included to calculate pathway costs. Proteins separated by a semicolon (;) are isoenzymes, the average of their costs is taken. Proteins separated with a comma (,) are consecutive enzymes in a pathways, and the sum of their costs (in C atoms) is taken.

Parameter	Value
$k_{cat,hxt}$	13.298 h^{-1}
$k_{cat,resp}$	0.051 h^{-1}
$k_{cat,rib}$	0.639 h^{-1}
$k_{cat,ferm}$	8.321 h^{-1}
$k_{cat,gly}$	1.804 h^{-1}

Table S3. Final module-k_{cat} values, as obtained after fitting the μ_{max} and the switch-point between respiration and fermentation to experimental data.

have found in literature, μ_{max}, of *S. cerevisiae* is $0.6h^{-1}$[12] . We fitted the absolute values of the module k_{cat}s such that at a fully saturating glucose concentration (glu $= 100000mM$), and with optimal (relative) enzyme allocation, a growth rate $\mu = 0.6h^{-1}$ is attained. Table S3 lists the so-obtained pathway k_{cat}s.

Next, we still had to determine the costs of the respiration pathway. This was not possible to obtain from literature, because the costs for mitochondria are difficult to estimate. Instead, we use these to "fit" the growth rate at which the switch from respiration to fermentation occurs to literature data. We used literature data for the growth rate where fermentation sets in to determine the pathway costs of respiration. Ethanol production in chemostats with *S. cerevisiae* sets in at a dilution rate between 0.25 and 0.28 h^{-1}.[13] We have assumed that in the middle of this interval ($d = 0.265\ h^{-1}$), an equal growth rate can be achieved using the fermentation pathway and the respiration pathway, when there is no ethanol present. The result of fitting the cost of the respiration module in this manner is that at dilution rates higher than $0.265h^{-1}$ the possibility for coexistence might arise. The pathway turnover number was divided by the cost of the respiration module to obtain the module-k_{cat} (Table S3).

S2.3.1 Comparison of model fit with literature data

As described above (section S2.3), we have fitted the model such that the μ_{max} and the switch-point between fermentation and respiration are consistent with literature data. While it is not our goal to make an as realistic model as possible, it is nonetheless instructive to compare additional model predictions with experimental data. Figure S1 compares predicted (A) residual glucose, (B) biomass yield (C) specific CO_2 production rate qCO_2 and (D) specific O_2 consumption rate qO_2 to literature of *S. cerevisiae* in chemostats. It is clear that the predictions are reasonably accurate. The offset in the specific CO_2 production flux is due of the fact that different strains switch at different dilution rates. Some strains maintain a high respiration flux when they start fermenting, and consequently, qO_2 does not decline with increasing D as the model predicts (figure S1D). In our model, specific fluxes are in units mmol L-cytosol^{-1} h^{-1}. We converted these to mmol gr-dry-weight^{-1}h^{-1} by multiplication with a factor 0.002, which corresponds to 2 ml cytosol per gram dry weight.[8] We converted the yield from gram biomass per millimole glucose to gram biomass per gram glucose.

S2.3.2 Sensitivity analysis of assumed parameters

We were not able to obtain or infer the affinities of the modules for ADP and ATP (i.e. $K_{M,\mathrm{gly,ADP}}, K_{M,\mathrm{resp,ADP}}$ and$K_{M,\mathrm{rib,ATP}}$). We have therefore arbitrarily set these to 1 mM (Table S1). To estimate the sensitivity of the model to these affinities, we performed a sensitivity analysis. We have set the affinity either 10 times higher or 5 times lower, and refitted the model. Although the values of the fitted model are different (Fig. S2A and S2B), the overall model behavior and area of coexistence

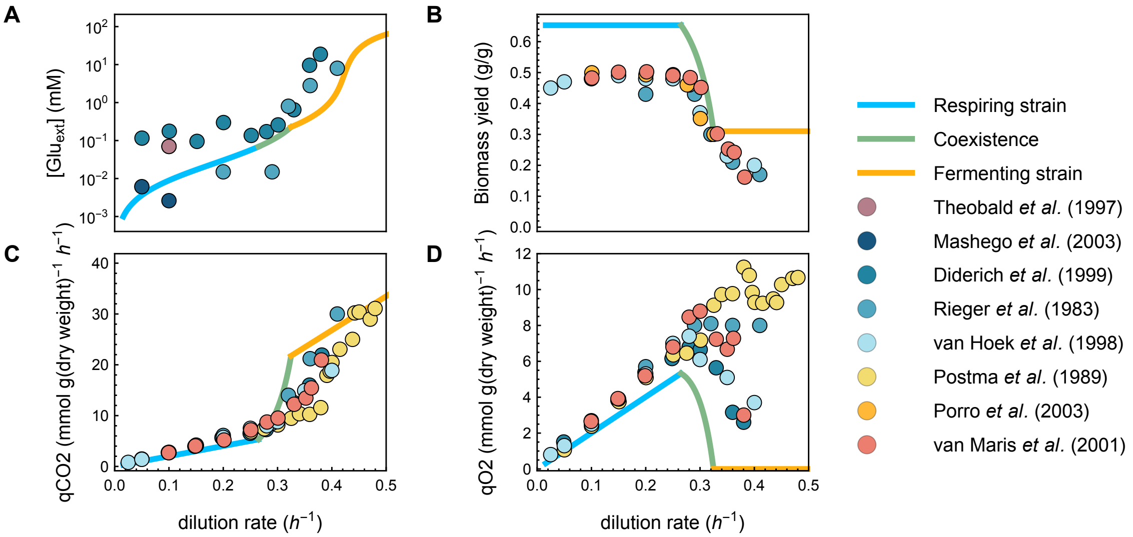

Figure S1. Comparison of the self replicator model of *S. cerevisiae* with literature data. Model predictions of the evolutionary steady state alongside literature data[6,13–19] for **A** residual substrate (on a logarithmic scale to distinguish the low substrate levels, (**B**) biomass yield, (**C**) specific CO_2 production flux and (**D**) specific O_2 uptake flux. The line color of the model predictions indicates whether the evolutionary stable state consists of a fully respiring strain (blue), a fully fermenting strain (yellow) or a coexistence of both strains (green).

are comparable (Fig. S2C). From this we conclude that the model behavior does not qualitatively depend on these values.

S2.4 Calculation of the optimal states in the chemostat

Here we describe how an evolutionary stable state of the chemostat can be calculated, for given dilution rate D and glucose feed concentration glu_{feed}. As a reminder, we define an evolutionary stable state as a state where the glucose, ethanol and biomass concentration are in steady state, and where no mutant with a different metabolic strategy (in terms of the enzyme concentrations) can invade.

The self-replicator model of *S. cerevisiae* discussed above allows us to calculate the growth rate given the glucose, ethanol and relative enzyme concentrations. In order to calculate the maximal growth rate of the cell for given glucose and ethanol concentration, we fix the ribosome flux and minimize the total amount of enzyme required to obtain that flux. The optimal strategy is always either purely respiratory or fermentative. However, it is also possible to calculate the optimal growth rate given a particular metabolic strategy. This is done by fixing $v_{resp}/(v_{resp}+v_{ferm})$, and calculating the minimal enzyme amount required to attain a certain v_{rib} given that constraint.

We distinguish three phases for optimal cells in the chemostat, related to the evolutionary stable state:

Phase I Below a dilution rate of $D = 0.265h^{-1}$ the respiring strategy outgrows the fermenting strategy, even at 0 mM of ethanol. Therefore respiring strains will always outcompete fermenting ones

Phase II Between $D = 0.265h^{-1}$ and $D = 0.358h^{-1}$, coexistence of a respiring and fermenting strain is potentially a evolutionary stable outcome. Whether this is indeed the case, depends on the glucose concentration in the feed.

Phase III Above $D = 0.358h^{-1}$, only fermentation: Since the maximal growth rate of respiring strains is $0.358h^{-1}$, at a dilution rate $D > 0.358h^{-1}$ no respiring strains can be present.

In phase I we can optimize the model with only a respiratory strain by minimizing total enzyme amount required to attain a certain rate of protein production. By doing this for a large number of glucose concentration, and interpolating the results, we obtain the (optimized) growth rate as function of the glucose concentration, $\mu_{\text{resp}}(\text{glu})$. Since in steady state it must hold that

$$\mu_{resp}(\text{glu}) = D, \tag{S17}$$

this can be used to calculate the steady state glucose concentration in the chemostat. The yield of a respiring stain is fixed, so this also fixes the total biomass density in the chemostat to:

$$X = Y_{X/S,\text{resp}} \left(\text{glu}_{feed} - \text{glu}\right) \tag{S18}$$

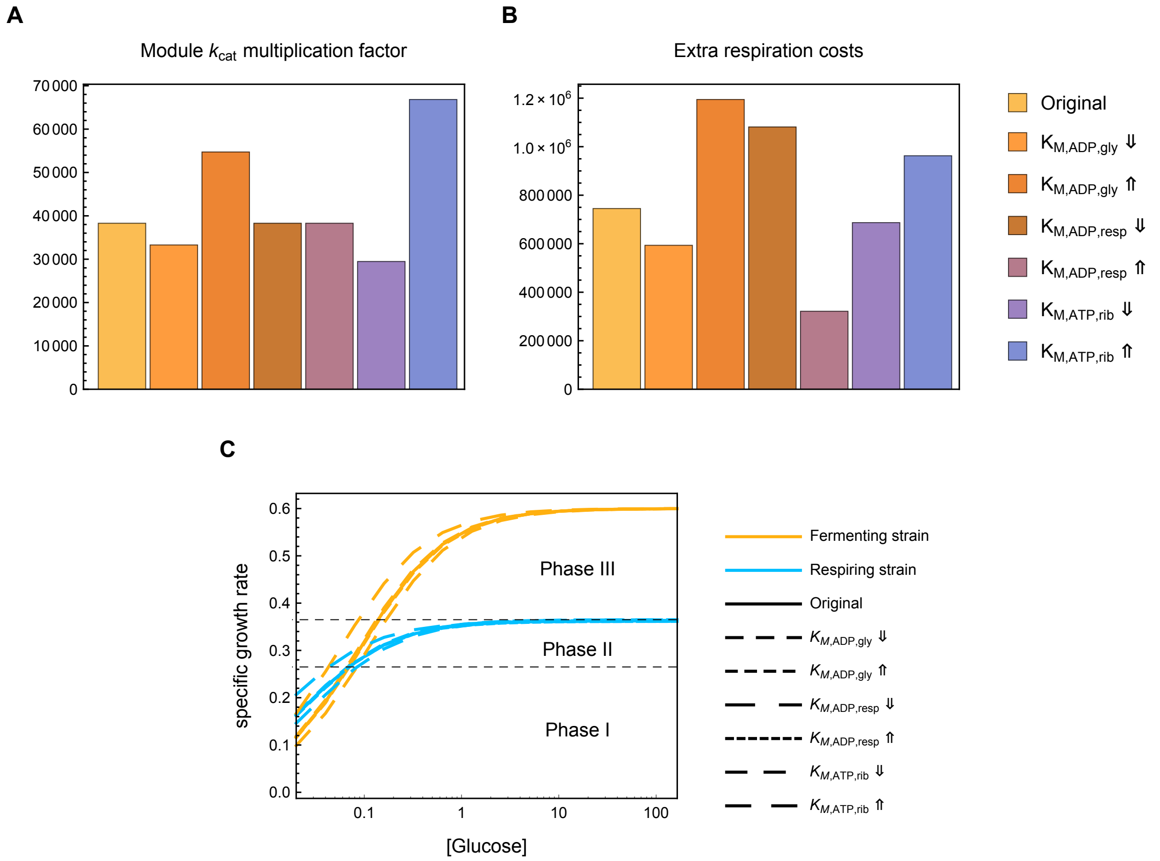

Figure S2. **Sensitivities of the affinities for ATP and ADP** A 5 times lower or 10 times higher affinity does effect the fitted parameters for the module k_{cat}s (**A**) and extra respiration costs (**B**), but the overall model behavior and range of coexistence remain comparable (**C**).

In phase II we can elucidate the evolutionary stable state by first calculating the glucose concentration at which the growth rate of the respiring strain equals the dilution rate, which is given by the solution to equation (S17). Next, we can calculate the ethanol concentration at which the fermenting strain has the same growth rate, at the glucose concentration calculated for the respirer by solving

$$\mu_{ferm}(\text{glu}, \text{EtOH}) = D \tag{S19}$$

for EtOH (using the value of glu found before). From the glucose feed concentration and the (known, fixed) ethanol yield unit per biomass produced by a fermenting cell, we can calculate the biomass density of the fermenting cells,

$$X_{\text{ferm}} = \text{EtOH}/Y_{\text{EtOH}/X}, \tag{S20}$$

and the respiring cells,

$$X_{\text{resp}} = Y_{X/S,\text{resp}} \left(glu_{feed} - glu - \frac{\text{EtOH}}{Y_{\text{EtOH}/X} \cdot Y_{X/S,\text{ferm}}} \right). \tag{S21}$$

If $glu_{feed} < glu + \frac{\text{EtOH}}{Y_{\text{EtOH}/X} \cdot Y_{X/S,\text{ferm}}}$ this equation does not have a (positive) solution. In that case the glucose feed concentration is too low for enough ethanol to accumulate to inhibit the fermenting strains to the extend that they grow at the same rate as the respiring ones, and no coexistence can occur.

In phase III, and in phase II when no coexistence is possible, we can calculate the ethanol concentration in the fermentor for a specific feed and residual substrate concentration, since the yield is fixed. With the substrate and ethanol concentration the growth rate of the fermenting strain can be calculated.

S2.5 Simulation of evolution in a chemostat

The general scheme of our simulation of evolution in a chemostat is similar to that used by Beardmore *et al.*[20] We restrict the possible phenotypes to $N = 50$ discrete metabolic strategies, where a metabolic strategy is defined by the relative flux through respiration: $v_{resp}/(v_{resp} + v_{ferm})$. The strategies are uniformly distributed between 0 (fully fermentative) and 1 (fully respiratory). We assume that, throughout the simulation, the enzyme concentrations of each phenotype are optimal given the constraint imposed by the strategy. This has both a pragmatic as well as a biological motivation. The biological motivation is that we are interested in the effect of metabolic strategy (fermentation vs. respiration) on fitness in the chemostat. This is best achieved assuming all else is optimal, because it avoids other confounding effects. Pragmatically, assuming all enzyme levels to be optimal given the constraint of the strategy greatly reduces the dimensionality of the simulation. It allows us to model 50 "species", whereas otherwise we would have to partition each of these into many other, one for each possible enzyme concentration configuration.

For each phenotype, we first define the function that relates the growth rate to the glucose and ethanol concentration. This is done by calculating the optimal μ given glu and EtOH for a number of different concentration in the range that is expected to occur during the simulation, and interpolating the result.

The dynamics are subsequently given by the following set of ODEs:

$$\frac{dX_i}{dt} = \varepsilon \sum_{j=1}^{N} (M_{ij} - \delta_{ij}) \cdot X_j + (\mu_i - D) \cdot X_i \qquad \forall i \tag{S22a}$$

$$\frac{d\text{glu}}{dt} = D(\text{glu}_{feed} - \text{glu}) - \sum_{i=1}^{N} \frac{\mu_i}{Y_{X,S,i}} \cdot X_i \tag{S22b}$$

$$\frac{d\text{EtOH}}{dt} = \sum_{i=1}^{N} \cdot Y_{\text{EtOH},X,i} \cdot \mu_i X_i \tag{S22c}$$

where δ_{ij} is the Kronecker delta, which is 1 when $i = j$ and 0 otherwise. These are equivalent to equation (S16) except for the term $\varepsilon \sum_{j=1}^{N} (M_{ij} - \delta_{ij}) \cdot X_j$, which describes the effect of mutations, modeled as a "flux" from one phenotype to another. M is the mutation matrix and each element M_{ij} the relative chance that a mutation in phenotype j gives rises to phenotype i. As such, $\varepsilon \sum_{j=1}^{N} M_{ij} X_j$ is that rate at which phenotype i arises by mutations. M_{ij} is assumed to be exponentially decaying with the "distance" between phenotypes, i.e. the difference in $v_{\text{resp}}/(v_{\text{ferm}} + v_{resp})$, while the columns of M need to sum to 1. This gives:

$$M_{ij} = \frac{e^{-|i-j|)/\tau}}{\sum_i e^{-|i-j|)/\tau}} \tag{S23}$$

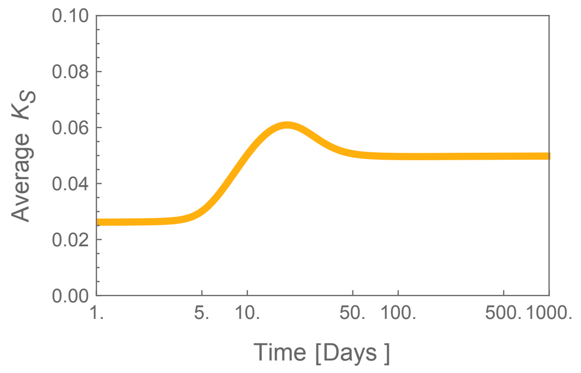

Figure S3. **Affinity of cells for substrate in evolutionary simulation** During the evolutionary simulation the average affinity of the cell first decreases (the Monod constant K_S increases) and then increases (the K_S decreases).

In the simulation of the evolution experiments the following parameters were used: $\varepsilon = 0.001h^{-1}$, $\tau = 0.05$, $\text{glu}_{feed} = 100$ mM and $D = 0.3h^{-1}$. The model was initialized with a single, mixed strategy present with X, glu and EtOH such that reactor was in steady state.

We calculate the average Monod constant (K_S) by fitting the growth data for each genotype with an Hill equation:

$$\mu = \mu_{max} \cdot \frac{[glu]^n}{[glu]^n + K_S{}^n} \tag{S24}$$

We take the μ_{max} by calculating the growth rate with a high glucose concentration and fit the Hill exponent n and the Monod constant K_S. The Hill exponents for the strains were between 1.27 and 1.66. With this data we calculated the average affinity of the cells over the course of the time simulation (Figure S3).

References

1. Dykhuizen, D. E. Selection in chemostats. *Microbiol. Mol. Biol. Rev.* **47**, 150–168 (1983).
2. Molenaar, D. *et al.* Shifts in growth strategies reflect tradeoffs in cellular economics. *Mol. Syst. Biol.* **5**, 323 (2009).
3. Neidhardt, F. C., Ingraham, J. L. & Schaechter, M. *Physiology of the bacterial cell: a molecular approach* (Sinauer Associates Sunderland, MA, 1990).
4. Famili, I., Forster, J., Nielsen, J. & Palsson, B. O. *Saccharomyces cerevisiae* phenotypes can be predicted by using constraint-based analysis of a genome-scale reconstructed metabolic network. *Proceedings of the National Academy of Sciences* **100**, 13134–13139 (2003).
5. Teusink, B. *et al.* Can yeast glycolysis be understood in terms of *in vitro* kinetics of the constituent enzymes? Testing biochemistry. *Eur. J. Biochem.* **267**, 5313–29 (2000).
6. Postma, E., Verduyn, C., Scheffers, W. A. & Van Dyken, J. D. Enzymic analysis of the Crabtree effect in glucose-limited chemostat cultures of *Saccharomyces cerevisiae*. *Appl. Environ. Microbiol.* **55**, 468–477 (1989).
7. Kresze, G.-B. & Ronft, H. Pyruvate dehydrogenase complex from baker' s yeast 1. Purification and some kinetic and regulatory properties. *Eur. J. Biochem.* **119**, 573–579 (1981).
8. van Heerden, J. H. *et al.* Lost in Transition: Startup of Glycolysis Yields Subpopulations of Nongrowing Cells. *Science* **343**, 1245114 (2014).
9. The-UniProt-Consortium. Activities at the Universal Protein Resource (UniProt). *Nucleic Acids Res.* **42**, D191–8 (2014).
10. Verschoor, a., Warner, J. R., Srivastava, S., Grassucci, R. a. & Frank, J. Three-dimensional structure of the yeast ribosome. *Nucleic Acids Res.* **26**, 655–661 (1998).
11. Warner, J. R. The assembly of ribosomes in yeast. *J. Biol. Chem.* **246**, 447–454 (1971).
12. Youk, H. & Oudenaarden, A. V. Growth landscape formed by perception and import of glucose in yeast. *Nature* **462**, 875–879 (2009).
13. van Hoek, P., van Dijken, J. P. & Pronk, J. T. Effect of specific growth rate on fermentative capacity of baker's yeast. *Appl. Environ. Microbiol.* **64**, 4226–4233 (1998).
14. Theobald, U., Mailinger, W., Baltes, M., Rizzi, M. & Reuss, M. In vivo analysis of metabolic dynamics in *Saccharomyces cerevisiae*: I. Experimental observations. *Biotechnology and bioengineering* **55**, 305–16 (1997).
15. Mashego, M. R., van Gulik, W. M., Vinke, J. L. & Heijnen, J. J. Critical evaluation of sampling techniques for residual glucose determination in carbon-limited chemostat culture of *Saccharomyces cerevisiae*. *Biotechnology and bioengineering* **83**, 395–9 (2003).
16. Diderich, J. A. Glucose Uptake Kinetics and Transcription of HXT Genes in Chemostat Cultures of *Saccharomyces cerevisiae*. *Journal of Biological Chemistry* **274**, 15350–15359 (1999).
17. Rieger, M., Käppeli, O. & Fiechter, A. The role of limited respiration in the incomplete oxidation of glucose by *Saccharomyces cerevisiae*. *Journal of general microbiology* **129**, 653–661 (1983).
18. Porro, D., Brambilla, L. & Alberghina, L. Glucose metabolism and cell size in continuous cultures of *Saccharomyces cerevisiae*. *FEMS Microbiology Letters* **229**, 165–171 (2003).
19. van Maris, A. J. A. *et al.* Modulating the distribution of fluxes among respiration and fermentation by overexpression of HAP4 in *Saccharomyces cerevisiae*. *FEMS yeast research* **1**, 139–49 (2001).
20. Beardmore, R. E., Gudelj, I., Lipson, D. A. & Hurst, L. D. Metabolic trade-offs and the maintenance of the fittest and the flattest. *Nature* **472**, 1–5 (2011).

Supplementary video. Simulation of evolutionary dynamics of an initial population of respiro-fermenters into two sub-populations: respirers and fermenters. Each phenotype is defined by the relative flux to fermentation (x-axis) and investment in transporter proteins (y-axis). First, fermenters appear and start to settle. These fermenters have an increased transporter expression relative to the initial respiro-fermenters. After about 15 days, respiro-fermenters with decreased transporter expression appear, which gradually become ever more respiratory until finally the respiro-fermenters are completely replaced. The maintenance of several genotypes close to the optimal strategy in the final state is the result of a mutation-selection balance. Details of the simulation can be found in the Supplementary Information S2.5.
